# Antiangiogenesis, Loss of Cell Adhesion and Apoptosis Are Involved in the Antitumoral Activity of Proteases from *V. cundinamarcensis* (*C. candamarcensis*) in Murine Melanoma B16F1

**DOI:** 10.3390/ijms16047027

**Published:** 2015-03-27

**Authors:** Dalton Dittz, Cinthia Figueiredo, Fernanda O. Lemos, Celso T. R. Viana, Silvia P. Andrade, Elaine M. Souza-Fagundes, Ricardo T. Fujiwara, Carlos E. Salas, Miriam T. P. Lopes

**Affiliations:** 1 Departamento de Farmacologia, Instituto de Ciências Biológicas, Universidade Federal de Minas Gerais, Av Antônio Carlos 6627, 31270-901 Belo Horizonte, Brazil; E-Mails: daltondittz@gmail.com (D.D.); cinthiafigueiredo@yahoo.com.br (C.F.); folemos@hotmail.com (F.O.L.); mtpl@icb.ufmg.br (M.T.P.L.); 2 Departamento de Fisiologia, Instituto de Ciências Biológicas, Universidade Federal de Minas Gerais, Av Antônio Carlos 6627, 31270-901 Belo Horizonte, Brazil; E-Mails: celsotarso@gmail.com (C.T.R.V.); andrades@icb.ufmg.br (S.P.A.); elainefagundes@gmail.com (E.M.S.-F.); 3 Departamento de Parasitologia, Instituto de Ciências Biológicas, Universidade Federal de Minas Gerais, Av Antônio Carlos 6627, 31270-901 Belo Horizonte, Brazil; E-Mail: fujiwara@icb.ufmg.br; 4 Departamento de Bioquímica, Instituto de Ciências Biológicas, Universidade Federal de Minas Gerais, Av Antônio Carlos 6627, 31270-901 Belo Horizonte, Brazil

**Keywords:** proteases, *V. cundinamarcensis*, antitumoral, melanoma B16F1, apoptosis, anchorage loss

## Abstract

The proteolytic enzymes from *V. cundinamarcensis* latex, (P1G10), display healing activity in animal models following various types of lesions. P1G10 or the purified isoforms act as mitogens on fibroblast and epithelial cells by stimulating angiogenesis and wound healing in gastric and cutaneous ulcers models. Based on evidence that plant proteinases act as antitumorals, we verified this effect on a murine melanoma model. The antitumoral effect analyzed mice survival and tumor development after subcutaneous administration of P1G10 into C57BL/6J mice bearing B16F1 low metastatic melanoma. Possible factors involved in the antitumoral action were assessed, *i.e.*, cytotoxicity, cell adhesion and apoptosis *in vitro*, haemoglobin (Hb), vascular endothelial growth factor (VEGF), tumor growth factor-β (TGF-β), tumor necrosis factor-α (TNF-α) content and *N*-acetyl-glucosaminidase (NAG) activity. We observed that P1G10 inhibited angiogenesis measured by the decline of Hb and VEGF within the tumor, and TGF-β displayed a non-significant increase and TNF-α showed a minor non-significant reduction. On the other hand, there was an increase in NAG activity. In treated B16F1 cells, apoptosis was induced along with decreased cell binding to extracellular matrix components (ECM) and anchorage, without impairing viability.

## 1. Introduction

During the last decades, a direct strategy has dominated the field of cancer therapy; the selected target was the tumor cell itself and any cytotoxic drug that destroyed tumor cells *in vitro* became a candidate for *in vivo* chemotherapy. However, it became apparent that tumor-killing drugs also target normal cells. Moreover, because of the inherent genetic instability of neoplastic cells, exposure to chemotherapy eventually results in selection of drug-resistant clones [1]. To avoid these effects, newer approaches involve a preliminary screening of drugs that target tumor-specific functions; *i.e.*, angiogenicity, loss of adhesion and/or migration and tumor antigenicity.

The significance of angiogenesis and metastasis for tumor development are well established and malignant cells in the melanoma microenvironment produce large numbers of angiogenic stimulators that enhance endothelial cell proliferation and migration of vascular endothelial growth factor (VEGF), and fibroblast growth factor (FGF-2) leading to vessel growth [2]. These factors are primarily controlled by hypoxia, certain cytokines and growth factors such as: tumor necrosis factor (TNF-α), transforming growth factor (TGF-β) and epidermal growth factor (EGF) that induce the expression of VEGF [3]. In addition to angiogenic cytokines, adhesion molecules, such as integrins (α_v_β_3_, α_v_β_5_, α_4_β_1_, and α_5_β_1_), are expressed in tumor cells and its microenvironment, modulating tumor initiation, progression, and angiogenesis [4].

P1G10 is a proteolytic fraction from *Carica candamarcensis* (*V. cundinamarcensis*), a member of *Caricaceae* family and common to many areas of South America. This fraction contains 14 proteinase isoforms, involved in plant protection against predators [5]. Interestingly, proteinases in *V. cundinamarcensis* display five to seven-fold higher activity compared to proteolytic enzymes from *C. papaya*, a probable consequence of an adaptive response to its habitat. The fraction induces skin epithelization after wound healing caused by dermoabrasions [6], burns [7], and gastric lesions [8]. P1G10 also displays fibrinogenolytic, fibrinolytic and antithrombotic activities, in a dose-dependent manner [9]. In most situations the biological activity of plant cysteine proteinases is explained by the hydrolytic activity, immunomodulation, and/or binding to circulating anti-proteases, upsetting the homeostasis of endogenous circulating proteinases [10]. Furthermore, the proteolytic mixture lacks significant dermal and systemic toxicity [6] and no mutagenic or genotoxic effects are evident [11]. In support of the enhanced wound healing activity, Gomes and coworkers [12,13] demonstrated that two of the proteinases in P1G10, (CMS2MS2 and CMS2MS3), display mitogenic activity on fibroblasts and other cell lineages, an effect mediated by the activation of MAP kinase pathway and which is independent of the proteolytic activity.

Several studies link the use of proteolytic enzymes to oncotherapy, [14,15,16]. Mice bearing Lewis lung carcinoma treated with proteolytic enzymes (trypsin, chymotrypsin and papain) showed increased survival and reduction of metastatic spread [14] and clinical studies show that individuals with mammary [17], ovarian, head and neck tumors [18], reduce the side effects of radiotherapy and/or chemotherapy and increase survival after proteinase adjunct treatment.

This evidence prompted us to evaluate the possible antitumoral effect of P1G10 using a B16F1 melanoma syngeneic model in C57BL/6J mice and likely mediators involved in its action.

## 2. Results and Discussion

In preliminary studies we characterized the chromatographic, electrophoretic profile and amidase activity of the P1G10 fraction. Herein, we analyze by mass spectroscopy the size of the antitumoral proteolytic fraction. One major peak of 25,481.6 Da and two minor peaks of 23,775.2 and 22,140.2 Da are shown in (Supplementary Figure S1). The smaller components match the size of proteolytic isoforms described earlier [5], and the larger peak (25,481.6) probably represents an unprocessed precursor usually present in latex preparations.

### 2.1. P1G10 Antitumoral Activity and Survival

**Figure 1:**
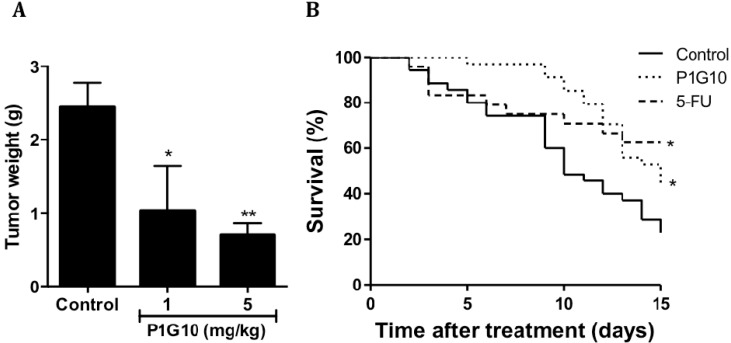
P1G10 antitumoral activity and survival in murine melanoma B16F1. (**A**) C57Bl6 mice (*n* = 40) were subcutaneously (*s.c.*) inoculated with B16F1 (5 × 10^5^ cells/100 µL), and treated with saline (control) or P1G10 1 or 5 mg/kg *s.c.*, for 15 days, * *p* < 0.05, ** *p* < 0.01, ANOVA, Dunnet’s post-test; and (**B**) The survival of animals with melanoma treated with saline (control), P1G10 5 mg/kg, *s.c.*, for 15-days or 5-FU (positive control), intraperitoneal *i.p*. * *p* < 0.05, Log-rank test was applied in survival experiments.

The experimental design to analyze the antitumoral activity involved three animal conditions: the control only injected with vehicle or, P1G10 at 1 or 5 mg/kg. The antitumor activity was evaluated on day 15th following treatment with P1G10, which initiated on day four, after B16F1 cell inoculation. The tumoral mass showed significant reduction in groups treated with 1 mg/kg (64%, *p* < 0.05), and 5 mg/kg (70%, *p* < 0.001) P1G10, relative to the control group (ANOVA Bonferroni post-test), as shown in Figure 1A.

Figure 1B shows that after eight days of treatment, the highest survival occurred in the group treated with P1G10 (97.1% survival), compared to the 5-FU group (70.8%), or the negative control (74.3%). The superior performance of the group treated with P1G10 lasted until the 12th day, when 69% of the animals were still alive. Beyond that period the survival of 5-FU and P1G10 became similar and both remained higher than the control during the remaining period. The Log-rank test applied to these data shows a statistical difference between both; the experimental group and the positive control, *versus* the untreated group, while a comparison between the experimental group and the 5-FU group showed no difference in survival.

Prior studies demonstrated that plant proteinases suppressed tumor growth, invasion and lung metastasis of murine melanoma [19]. Bromelain was reported to increase survival in P-388 leukemia, sarcoma (S-37), Ehrlich ascites tumor and Lewis lung carcinoma but not in melanoma B16F10 [20], while the proteinase fastuosain (*Bromelia fastuosa*), reduced the number of lung metastatic nodules in mice [21]. As seen, the therapy using plant proteinases exhibits antitumoral efficacy between 1 and 50 mg/kg (2–65 nMol/dose) while conventional chemotherapy utilizes higher doses 5–40 mg/kg (0.2–5 μmol/dose); this 1000-fold difference implies an increased chance of undesired effects during chemotherapy [22,23,24]. While these enzymes belong to the same C1 family, bromelain and fastuosain (Bromeliaceae) share closer structures than proteinases in P1G10, which rank closer to papain (Caricaceae) [7,25]. On the other hand, bromelain is an acid proteinase while every isoform in P1G10 is highly basic [5,26].

Using PFLNA as substrate the enzymes composing P1G10 display *k_cat_*/*K_m_* between 57,407 and 375 M^−1^·s^−1^ while the various bromelain fractions show *k_cat_*/*K_m_* between 0.00975 and 0.00038 M^−1^·s^−1^; in other words the catalytic efficiency of P1G10 is at least 4 × 10^4^ higher than bromelain [7,27]. In summary, the structural and kinetic differences between P1G10 and bromelain-like enzymes do not rule out the existence of a common antitumoral mechanism. 

### 2.2. Tumor Angiogenic and Anti-Inflammatory Parameters and P1G10 Content in Tumor Tissue

**Figure 2:**
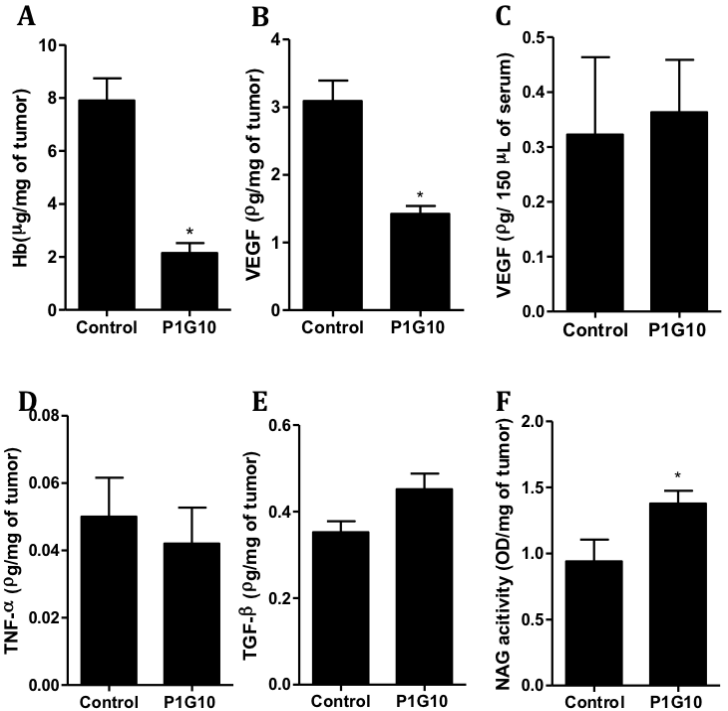
Angiogenic and inflammatory parameters in melanoma B16F1 animals treated with P1G10. Tumor tissue from animals (*n* = 20) treated with P1G10 5 mg/kg or the control was processed and analyzed as described in Methods Section. Tumoral: (**A**) Hemoglobin (Hb); (**B**) Vascular endothelial growth factor (VEGF); (**D**) Tumor necrosis factor-α (TGF-α); (**E**) TGF-β; (**F**) *N*-acetyl-glucosaminidase (NAG) activity; Serum: (**C**) VEGF, * *p* < 0.01, Student’s *t* test.

The effect of P1G10 (5 mg/kg) on cell and/or angiogenic markers (Hb, TNF-α, TGF-β, VEGF and NAG activity) was evaluated in tumor tissue, but, VEGF was also measured in serum samples from tumor bearing animals. Figure 2A shows reductions in tumoral Hb (73%) and VEGF (54%) (Figure 2B) compared to the control group (*p* < 0.01, Student’s *t* test). However, similar VEGF levels were found in serum samples from tumor treated animals relative to untreated tumor bearing controls (Figure 2C). In addition, Figure 2D,E; show that TNF-α and TGF-β levels were not significantly altered compared to their controls. On the other hand, the NAG activity in tumors showed a significant increase (47%, *p* < 0.01, Student’s *t* test), following P1G10 treatment (Figure 2F).

**Figure 3:**
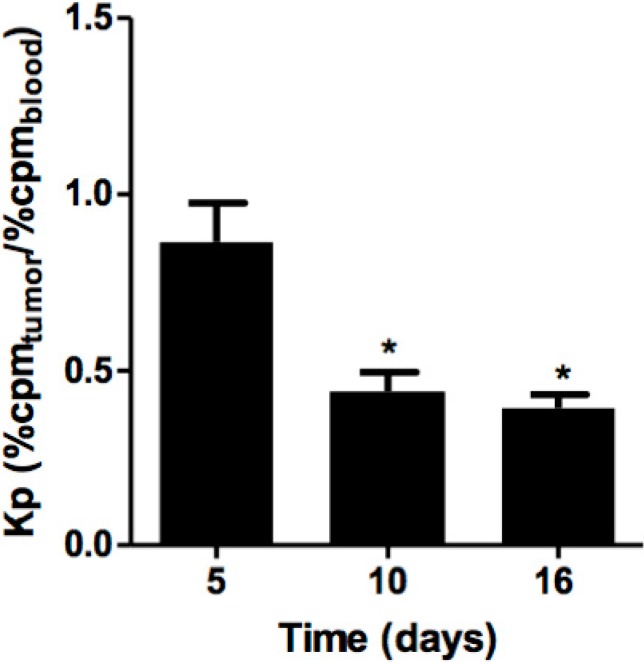
Distribution of ^99m^Tc-labeled P1G10 in B16F1 tumors. C57BL6 mice (*n* = 30) bearing B16F1 melanoma were treated daily with P1G10. Radiolabeled ^99m^Tc-P1G10 was included with the last treatment, 1 h before dissection. Tumors were removed and blood collected and radioactivity measured in ANSR γ-counter. * *p* < 0.05 compared to the five-days group, ANOVA, Bonferroni’s post-test.

The distribution of P1G10 in tumoral tissue during treatment was evaluated in the presence of P1G10 previously labelled with ^99m^Tc. B16F1 melanoma-bearing mice were divided into three-interval groups; 5th, 10th and 16th days, treated with 5 mg/kg P1G10 daily, and with the last treatment, each group received a similar dose containing ^99m^Tc-P1G10, as well. The radioactive uptake in each tumor was corrected by variations of the total radioactivity received by each animal. The distribution of ^99m^Tc-P1G10 was expressed as a partition coefficient (Kp) between tumor and blood. The Kp values were corrected by the mass variation among tumors. Figure 3 shows a significant reduction of Kp at the 10th (48.9%, *p* < 0.05) and 16th days (54.4%, *p* < 0.05) relative to day five, ANOVA, Bonferroni’s post-test. To account for possible changes in blood and organ distribution of P1G10 influenced by tumor, separate control experiments were conducted to assess the distribution of ^99m^Tc-P1G10 in normal and tumor bearing animals. The results of these experiments confirmed that there were no changes in blood and organ distribution between normal and tumor bearing animals (results not shown).

In previous studies, it was demonstrated that 0.1% P1G10 (*w*/*v*) induced angiogenic parameters in dorsal sponge-discs implanted in a non-tumor rodent model [8]. Considering the relevance of angiogenesis during tumor development [28], we measured the influence of P1G10 on angiogenesis using the B16F1 model. The four-fold reduction of hemoglobin observed in tumor tissue contrasted with the increase observed earlier in the sponge regenerating model and must be attributed to a direct or indirect discriminating effect by P1G10 between tumor and normal cells (Figure 2A). In similar experiments we demonstrated that the angiogenic parameters stimulated by P1G10 on the sponge implant was reverted when an Ehrlich tumor develops in the same animal bearing the implanted sponge (unpublished data). We further assessed tumor VEGF levels in animals treated with P1G10 and demonstrated that they are significantly reduced (Figure 2B). VEGF is a cytokine that stimulates blood vessel formation and is secreted by both normal and tumor cells; however, it is usually over-expressed in tumors [29]. Also, while VEGF is reduced by about 50% in the tumor sample following P1G10 treatment, serum VEGF within the same animal was non-significantly changed, compared to serum levels in untreated animals (control 0.32 ± 0.14; P1G10 5 mg/kg 0.52 ± 0.18) (Figure 2C), suggesting that the action of P1G10 is localized to the tumor. A confirmation of these data came from *in vivo* distribution experiments with ^99m^Tc labelled P1G10. Figure 3 shows a progressive decrease of ^99m^Tc-P1G10 within the tumor between 5 and 15 days, suggesting an antiangiogenic effect. Regardless of this effect, the biodistribution of P1G10 in non-tumoral tissues was similar in non-tumor and tumor bearing animals (data not shown).

The ambiguous role of P1G10 increasing angiogenic parameters during wound healing and reducing these parameters in tumor tissue can be ascertained if considering that wound healing and tumorigenesis bear common cellular and molecular mechanisms [30]. Alexander Haddow suggested that “tumor production results from wound overhealing” [31] Based on this hypothesis, the effect(s) of P1G10 must be located at a site(s) during healing process before initiation of tumor specific reactions and that this “action site” is repressed under influence of P1G10.

TGF-β measured in this model depicted a moderate (28.2%) non-significant increase compared to the tumor control without P1G10. The TGF-β role is unclear; some evidence assigns a tumor growth suppressive function in certain malignancies, while others link the increase in TGF-β expression following gene transfection to angiogenesis in prostate carcinoma [32,33]. It seems that the role of TGF-β depends on the cellular context in which the cytokine is acting [34]. Patients suffering a variety of ailments (rheumatoid arthritis, herpes zoster or diabetes) treated with a proteinase cocktail including bromelain and papain, trypsin and chymotrypsin exhibit serum TGF-β decrease, but it is unclear if one of these enzymes or the mixture was responsible for the effect. The authors attribute this reduction to an effect on the proteinase inhibitor α2 macroglobulin that binds to and inhibits TGF-β [35,36]. We did not detect any TGF-β serum changes on animals treated with P1G10 when compared to healthy animals (data not shown).

The minor non-significant decrease of tumoral TNF-α (Figure 2D) is an unlikely explanation of the decreased angiogenesis; on the contrary, it was demonstrated that increased TNF-α promoted local selective destruction of tumor blood vessels. On the other hand, TNF-α chronically produced, acts as potent tumorigenic, contributing to tissue remodeling, stromal development, and inducing transcription of VEGF mRNA, thus promoting angiogenesis [37]. Similarly, in a non-tumoral human inflammatory condition, bromelain decreased expression of TNF-α and other inflammatory mediators [38]. Again, it is not clear which of the multiple roles attributed to TNF-α applies to the B16F1 model.

Among cells infiltrating tumor stroma, macrophages are of particular importance [37,39] by secreting pro-inflammatory cytokines and growth factors (VEGF and endothelin-2) that favor tumor progression [30,40]. By measuring NAG, a lysosomal enzyme produced by macrophages [41], we observed a significant increase (32%) in the P1G10 treated group (Figure 2F). Engwerda and co-workers showed that bromelain, a cysteine proteinase, also activates macrophages *in vitro* and *in vivo* [42], while levels of pro-angiogenic cytokines, TGF-β and TNF-α, showed non-significant changes by P1G10. We hypothesize that the reduction in intratumor VEGF was in part a consequence of the decrease in the number of tumoral cells, the largest producers of this cytokine [29].

### 2.3. Cytotoxicity and Cell Apoptosis

As shown in Table 1, the inhibitory concentration (IC_50_) by P1G10 was similar in normal (CHO, CIP, BHK-21) and tumoral cell lines (4T1, Ehrlich, B16F1). These values ranged between 12.7 and 28.7 µg/mL and the lowest IC_50_ corresponded to tumoral cells.

**Figure 4:**
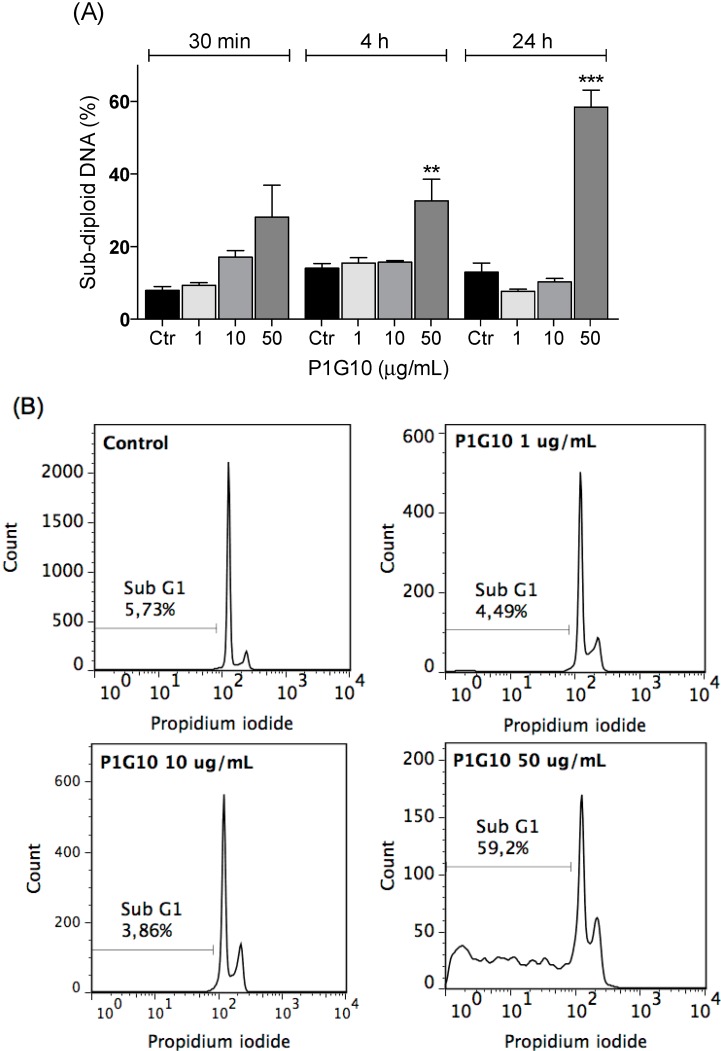
Cell Death. (**A**) B16F1 cells were treated with P1G10 (1, 10 or 50 µg/mL) for 30 min, 4 or 24 h. At the end of treatment, cells were resuspended in hypotonic fluorochrome solution containing propidium iodide. After 2–4 h at 4 °C in the dark, the fluorescence of individual nuclei was measured using a FACS flow cytometer and DNA fragmentation was analyzed by FlowJo Software. Data shown are mean ± SD; and (**B**) Representative histograms are shown of sub-diploid content after P1G10 treatment for 24 h. ** *p* < 0.01; *** *p* < 0.001, ANOVA, Bonferroni’s post-test.

**Figure 5:**
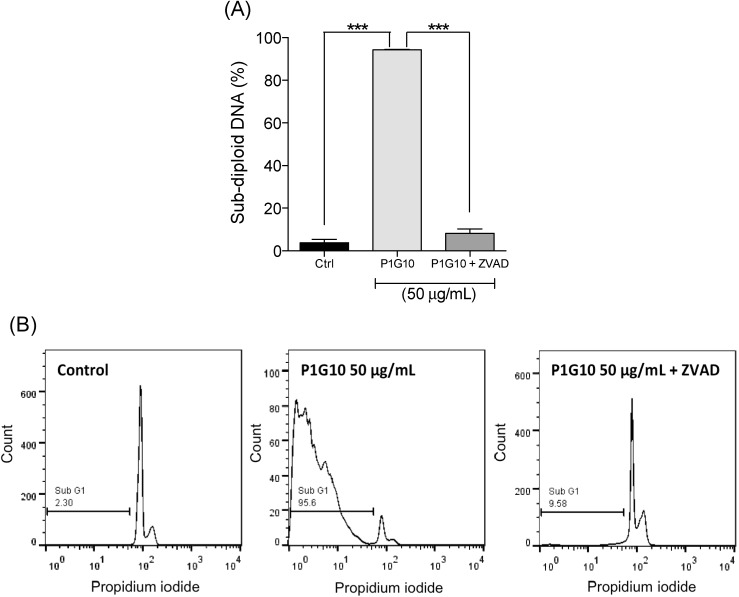
Caspase involvement in cell death. (**A**) B16F1 cells were pre-incubated with 40 μM ZVAD-FMK 40 min before treatment with 50 μg/mL P1G10 for 24 h. Propidium iodide was added and nuclear fluorescence scored in a FACS flow cytometer. DNA fragmentation was analyzed using FlowJo Software. Data are shown as mean ± SD and (**B**) Representative histograms of the sub-diploid content after P1G10 treatments in the presence or absence of ZVAD-FMK are shown (*** *p* < 0.001, ANOVA, Bonferroni’s post-test).

The pro-apoptotic potential of P1G10 was analyzed in supernatants of B16F1 cultures. Cellular DNA was quantitated by flow cytometry with propidium iodide. While no changes in sub-diploid DNA were observed at 1 or 10 µg/mL P1G10, at 50 µg/mL there was an increase in sub-diploid DNA (DNA fragmentation) after 4 h (2.3-fold, *p* < 0.01) or 24 h exposure (4.5-fold, *p* < 0.001, ANOVA, Bonferroni’s post-test), compared to the control (Figure 4). In addition, DNA fragmentation induced by P1G10 50 μg/mL was inhibited by pre-treatment with caspase inhibitor ZVAD-FMK, as shown in Figure 5. In similar studies, bromelain enhanced the expression of apoptotic activators p53 and Bax in mouse skin papilloma [43] and an aqueous extract of *Carica papaya* whose main component is papain induced apoptosis in acute Jurkat cells, used as model to study acute lymphocytic leukemia [44].

**Table 1:** Cytotoxicity of P1G10 fraction (IC_50_) in normal and tumor cell lines.

Types	Cell Lines	IC_50_ (µg/mL)
Tumoral	B16F1	12.7
	4T1	9.1
	Ehrlich	6.9
Normal	CHO	28.7
	CIPs	16.6
	BHK-21	19.0

Cells were exposed for 72 h to increasing concentrations of P1G10, and cell viability was determined by MTT metabolism. The IC_50_ was determined after calculating the regression of experimental data (*r* ≥ 0.90). Data shown are representative of three independent experiments. Tumoral cell lines: B16F1—murine nonmetastatic melanoma, 4T1 and Ehrlich—murine breast carcinomas. Normal cell lines: CHO—Chinese hamster ovary normal, CIPs—rabbit aorta endothelial cells and BHK 21—Chinese hamster kidney.

### 2.4. Loss of Adhesion on Polystyrene Plates or Extracellular Matrix Components (ECM) by B16F1 Melanoma Cells

**Figure 6:**
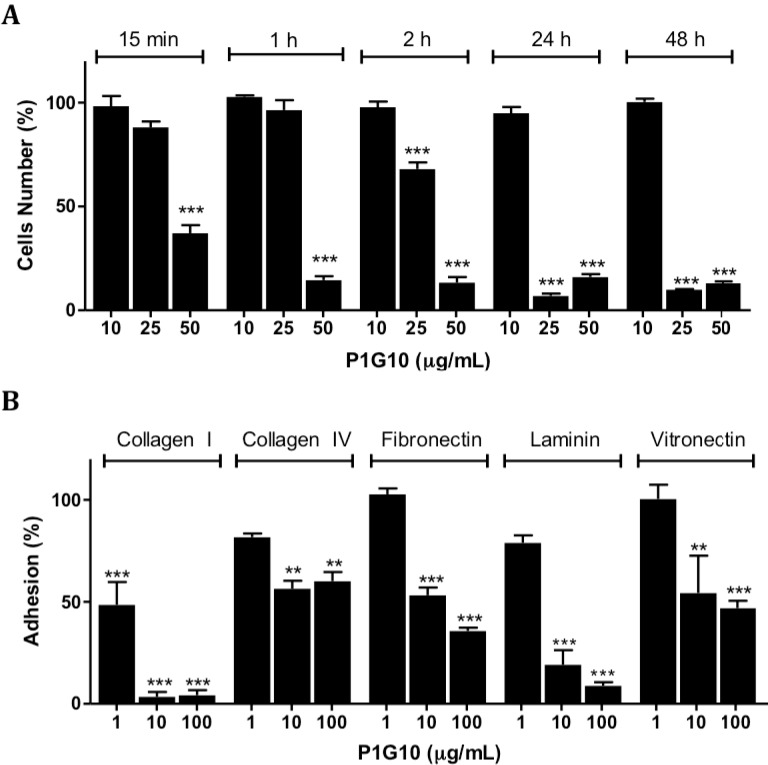
Cell adhesion analysis. (**A**) Cells were exposed to P1G10 (1–100 µg/mL) during various intervals up to 48 h. The absorbance was measured at 570/600 nm; and (**B**) Treated cells were plated on wells coated with extracellular matrix components. After 1 h incubation adhered cells were stained and scored at 540 nm. Data are shown as mean ± SD. For details see the Method Section. ** *p* < 0.01; *** *p* < 0.001, ANOVA, Bonferroni’s post-test.

The effect of P1G10 on cell adhesion was initially studied on polystyrene plates. Figure 6A shows no change in adhesion of B16F1cells between 15 min and 48 h at 10 µg/mL P1G10, while at 50 µg/mL P1G10, a 63% reduction was evident by 15 min and became stronger (≥83%) at subsequent intervals (*p* < 0.001, ANOVA, Bonferroni’s post-test). Loss of adhesion at 25 µg/mL P1G10 became significant at 2 h (32%) and later at 24 and 48 h, it became similar to the higher dose (92%) (*p* < 0.001, ANOVA, Bonferroni’s post-test). There was no difference in viability of P1G10 treated cells in suspension compared to the untreated group, suggesting that cell competence was not affected (data not shown).

The ability of B16F1 cells to adhere onto different extracellular matrix components (ECM) after exposure to P1G10 was studied using Millicoat Screening kit (Millipore, Billerica, MA, USA). Cells were exposed to 100 µg/mL P1G10 and then seeded onto plates coated with collagen I, collagen IV, fibronectin, laminin or vitronectin. The attached cells were stained with crystal violet and quantified by spectrophotometry at 540 nm. Figure 6B displays the percent adhesion data, expressed relative to the control, arbitrarily assigned 100%. The results show that P1G10 impaired attachment of B16F1 cells with each ECM component at 10 and 100 µg/mL (*p* < 0.01 and *p* < 0.001, respectively, ANOVA, Bonferroni’s post-test). The effect was stronger for laminin and collagen I. Cell adhesion to collagen I became evident at 1 µg/mL of P1G10, while binding to collagen IV was significant at ≥10 µg/mL (*p* < 0.001, ANOVA, Bonferroni’s post-test).

In many stages during tumor progression the interactions between cell–surface–adhesion molecules and ECM, and/or cell–cell interaction suffer changes [44,45]. In B16F1 cells on plastic support, morphological changes occur upon a short incubation period (15 min) with P1G10 (10 μg/mL), leading to rounding and desquamation, in a time- and concentration-dependent manner (data not shown). The largest decrease was observed with collagen I, followed by laminin, at each assayed concentration. Although several glycoproteins are surface molecules interacting with ECM, most cell adhesion comprises binding of α_4_β_1_ integrins to collagen I, which is the main component of ECM mediating melanoma cell attachment [46,47,48]. The role of proteinases on cell surface components was demonstrated; in the case of bromelain, 14 surface molecules associated with leukocyte adhesion were removed [49,50,51]. Novak and Trnka also proposed that a mixture of trypsin and chymotrypsin promoted morphological changes in B16F10 melanoma cells, probably by their action on cadherins [52]. It is likely that P1G10 removes cell surface components from B10F1 cells explaining the loss of adherence observed.

The relationship between loss of cell adhesion and apoptosis observed with P1G10 in Figure 5 and Figure 6 is termed anoikis [53,54,55]. The loss of adhesion promotes a disruption of cytoskeleton, culminating with the release of cytochrome C and induction of cell death [53]. The pro-apoptotic effect of P1G10 is mediated by caspases, since the use of a nonspecific caspase inhibitor (ZVAD-FMK) reduced the number of dead cells treated with P1G10. These data support the possible connection between loss of cell adhesion and subsequent death, resulting from P1G10. Interestingly, bromelain at 1 mg/kg promoted DNA fragmentation of tumor bearing animals and *C. papaya* papain at 10–20 mg/mL induced apoptosis in Jurkat cultured cells [56].

## 3. Experimental Section

### 3.1. Animals

Male C57BL/6J mice (18–25 g), obtained from the Centro de Bioterismo (CEBIO/ICB/UFMG), were housed at 22 ± 2 °C under a 12/12 h light/dark cycle with free access to food and water were used. The animals were acclimatized to the laboratory environment for at least 24 h before assays. All animal handling and experimental procedures were conducted with prior approval by the Ethics Committee in Animal Experimentation at the Federal University of Minas Gerais (Process n.103/2007).

### 3.2. Production of P1G10

The isolation of P1G10 was described previously [8]. Briefly, freeze-dried latex was dissolved and incubated at room temperature in buffer containing 25 mM l-cysteine, 5 mM DTT and 10 mM EDTA pH 5.0 in 1 M sodium acetate solution. The suspension was centrifuged at 4000× *g* 10 min at room temperature and the filtered supernatant (Whatman #1, Wilmington, MA, USA) was chromatographed through Sephadex G-10 (25 mm × 400 mm) previously equilibrated with 1 M sodium acetate pH 5.0 at room temperature. The first protein fraction (P1G10) containing the bulk of proteolytic activity was concentrated by ultrafiltration (10,000 Da pore size) and stored at −20 °C until use. The recovered sample was standardized by its proteolytic activity, SDS-PAGE electrophoresis and HPLC.

Matrix-Assisted Laser Desorption/Ionization Mass Spectrometry (MALDI-MS) was used to characterize the molecular mass of proteolytic components present in P1G10. Samples (25 µg) were mixed with α-cyano-4-hydroxy-cinnamic acid (matrix) dissolved in 0.1 g/100 mL trifluoroacetic acid and processed in a UltrafleXtrem MALDI-TOF mass spectrometer (Bruker Daltonics, Leipzig, Germany), using Protein Calib Standart 2 (Bruker Daltonics) as standard.

### 3.3. Cell Culture

The B16F1 cell line, low-metastatic melanoma, was a gift from the Instituto Ludwig de Pesquisa sobre o Câncer (Sao Paulo, Brazil); CHO (Chinese ovary hamster) and BHK 21 (Baby hamster kidney) cells were gifts from the Pan-American FMD Center, Rio de Janeiro, Brazil. CIPs (rabbit aorta endothelial) and Ehrlich cell lines were courtesy of Helena Nader, Biochemistry and Immunology, Federal University of São Paulo, Brazil and Geovane Cassali, Pathology, Federal University of Minas Gerais, respectively. The 4T1 cell line was obtained from the American Tissue and Cell Collection, USA. B16F1, 4T1, Ehrlich, CHO and BHK21 cells were grown in RPMI medium (GIBCO, Carlsbad, CA, USA) and CIPs were grown in F12-HAM (GIBCO). The media were supplemented with 10% heat-inactivated fetal bovine serum (FBS) (GIBCO), 1% penicillin/streptomycin (GIBCO) and cultured at 37 °C in a humidified 5% CO_2_ atmosphere.

### 3.4. B16F1 Melanoma Model

A murine melanoma model was established according to Zuberek and co-workers [57]. Briefly, B16F1 cells (5 × 10^5^ cells/100 µL) were subcutaneously (*s.c.*) inoculated into the right flank of C57BL/6J anesthetized mice, as recommended by the NCI Division of Cancer Treatment and Diagnosis (DCTD), USA for evaluation of new antineoplastic agents. The cervical *s.c.* injection of P1G10 started four days after tumor cell inoculation.

### 3.5. Antitumor Activity

Animals bearing B16F1 melanoma were *s.c.* daily treated with P1G10 (1 or 5 mg/kg) or saline (control). After 15-days treatment, mice were sacrificed in a CO_2_ chamber and the tumoral mass measured.

### 3.6. Survival

The animal survival was evaluated as described by Baez and coworkers [20]. Animals with tumor were treated for 15 days with P1G10 5 mg/kg or saline, *s.c.*, daily, or 5-FU 20 mg/kg, *i.p.* on days one, five and 10. After treatment, the animals were kept under a dark/light cycle with food and water *ad libitum*. The deaths were scored daily during the fifteen days after treatment was completed.

### 3.7. P1G10 Levels on Tumor

The evaluation of P1G10 levels in the tumor was done with ^99m^Tc-labeled P1G10, according to Lemos and coworkers [6]. The assay conditions were similar to that described for the antitumoral activity, except that during the last treatment the animals received ^99m^Tc-P1G10 (5.4 × 10^6^ cpm/mg protein) 5 mg/kg. The mice were sacrificed at different intervals (5th, 10th or 16th days). The radioactive distribution in tumor, blood and specific organs was measured in a γ-counter (Nuclide^TM^ TH 22, Mediso, Hungary).

### 3.8. Tumor Hemoglobin Concentration and N-Acetyl-β-d-glucosaminidase activity (NAG)

These experiments were carried out according to Plunkett and Hailey [58]. To 100 mg of tumor tissue from animals treated with 5 mg/kg P1G10 or saline, as described above, 2 mL Drabkin reagent was added and homogenized in an ultra-turrax mixer. The homogenate was centrifuged for 40 min, at 10,000 rpm and the supernatant filtered through a 0.22 µm Millipore filter. Aliquots of supernatant (100 µL) were added to a 96-well plate, and hemoglobin was quantified at 540 nm. The NAG activity was determined using the sediment previously obtained. A 0.9% NaCl solution containing 0.1% (*v*/*v*) Triton X-100 was added to suspend the sediment, mixed briefly and centrifuged for 10 min at 3000 rpm, 4 °C. One-hundred µL of the supernatant was added to 100 µL of *p*-Nitrophenyl-N-acetyl-β-d-glucosaminide dissolved in 0.1 M citrate/sodium phosphate buffer, pH 4.5 in a 96-well plate and incubated for 10 min at 37 °C, and 100 µL 0.2 M glycine, pH 10.6 buffer was added to stop the reaction. The enzyme activity was determined spectrophotometrically at 405 nm using an ELISA reader and expressed as OD/mg tumor [40].

### 3.9. Cytokines Quantification

Sample supernatant (100 µL) obtained as described above, or serum (150 µL) were mixed with 500 µL PBS pH 7.4 plus 0.05% Tween-20 and centrifuged for 30 min at 12,000× *g*, 4 °C and the supernatant recovered for cytokine assay. Cytokines (VEGF, TNF-α, TGF-β) were determined by immunoassay using anti-mice DuoSet^®^, R&D Immunoassay Kit (Minneapolis, MN, USA), as described by Teixeira and coworkers [59].

### 3.10. Cytotoxicity Activity of P1G10

The cytotoxic effect of P1G10 cells was evaluated by the MTT (3-(4,5)-dimethylthiahiazo(-z-y1)-3,5-diphenytetrazoliumromide) assay [59]. Briefly, exponentially growing cells, seeded in 96-well plates (10^3^ cells/well), were incubated with different concentrations (0.01–100 µg/mL) of sterile P1G10 for 72 h. At the end of this period, 10 µL of 5 mg/mL MTT (Sigma Chemical Co, St. Louis, MO, USA) was added onto each well and incubated at 37 °C with gentle stirring for 4 h. The cells were then lysed with dimethyl sulfoxide (DMSO) and the absorbance determined at 540 nm using an ELISA reader.

### 3.11. Flow Cytometer Analysis

DNA fragmentation as indicator of apoptosis was assessed by cell cycle analysis of total DNA content, as described by Nicoletti and coworkers [60], with slight modifications. B16F1 cells were exposed to P1G10 (1–100 μg/mL) or RPMI 1640 plus 10% FBS (control) up to 48 h at 37 °C in 5% CO_2_ humidified atmosphere. To evaluate the role of caspases on DNA fragmentation, 40 μM ZVAD-FMK were added to the culture 40 min before P1G10. A total of 2 × 10^5^ cells detached from plates were suspended in 0.3 mL hypotonic fluorochrome solution containing 50 µg/mL propidium iodide and 0.1% Triton X-100 in 0.1% sodium citrate. After 2–4 h incubation at 4 °C in the dark nuclei fluorescence was measured in a FACS flow cytometer (Becton-Dickinson, Mountain View, CA, USA).

### 3.12. Cell Adhesion Assays

Adhesion to polystyrene plates. B16F1 cells were seeded at a density of 2 × 10^3^ cells/100 µL/well in uncoated plates and incubated at 37 °C in 5% CO_2_ humidified atmosphere. After 24 h, cells were exposed to P1G10 (10, 25 or 50 µg/mL) or control solution (RPMI 1640 plus 10% FBS) for up to 48 h at 37 °C in 5% CO_2_ humidified atmosphere. Cells that remained attached were rinsed twice with PBS and 100 µL of 10 µg/mL resazurin dissolved in RPMI 1640 supplemented with 10% FBS, was added.

Adhesion to plates coated with extracellular matrix components (ECM), CytoMatrix™ Cell Adhesion Strips, Chemicon^®^ (Darmstadt, Germany). A suspension of B16F1 cells at 5 × 10^5^ cells/mL was mechanically dissociated and exposed to P1G10 (1 to 100 μg/mL) or RPMI 1640 plus 10% FBS (control) for 2 h. Then, 100 µL of cell suspension were seeded onto ECM coated plates and incubated at 37 °C in 5% CO_2_ humidified atmosphere for 1 h. The adherent cells were stained with crystal violet as recommended by the manufacturer and quantified at A_540_ with an ELISA reader. The results were expressed as percentage adhesion of treated cells relative to untreated control cells (assigned 100% adhesion) after blank subtraction.

## 4. Conclusions

These findings show that the antitumoral effect on murine B16F1 melanoma by P1G10 results in tumor mass reduction and survival increase similar to that observed with 5-FU. We hypothesize that these findings are linked to reduced angiogenesis resulting from decreased VEGF. It is unclear whether these effects arise as consequence of the loss of adhesion and or apoptosis observed. Finding a group of proteinases that induce epithelization of normal tissue and at the same time inhibit tumoral growth tissue is unique, and may be used to probe pathways relevant to cell regeneration and tumorigenesis.

## Supplementary Materials

Supplementary materials can be found at http://www.mdpi.com/1422-0067/16/04/7027/s1.
