# Supplementary material for: Antiangiogenesis, Loss of Cell Adhesion and Apoptosis Are Involved in the Antitumoral Activity of Proteases from V. cundinamarcensis (C. candamarcensis) in Murine Melanoma B16F1
Source: Int J Mol Sci. 2015 Mar 27;16(4):7027–44. doi: 10.3390/ijms16047027 (PMC4425002; doi:10.3390/ijms16047027)
Supplement: Supplementary file 1 [file ijms-16-07027-s001.pdf]

Supplementary Information

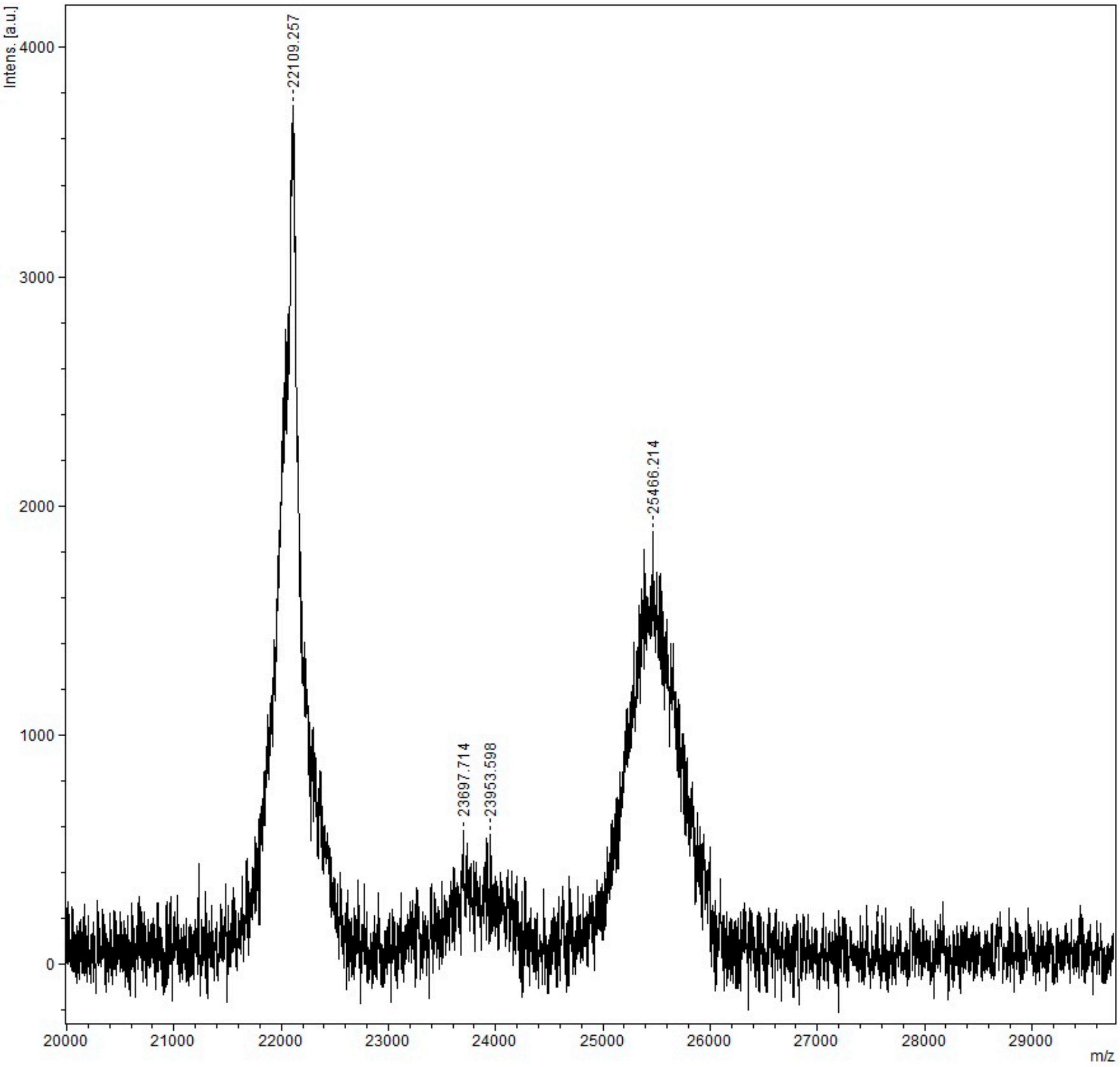

Figure S1. Mass spectrometric analysis of P1G10. The proteolytic fraction was desalted by passage on C4 HPLC column (Vidac). The absorbing fraction from HPLC was mixed with α-cyano-4-hydroxy-cinnamic acid (matrix) dissolved in 0.1 g/100 mL trifluoroacetic acid and processed in a UltrafleXtrem MALDI-TOF mass spectrometer (Bruker Daltonics, Leipzig, Germany).
